# High Accuracy Ultraviolet Index of Refraction Measurements Using a Fourier Transform Spectrometer

**DOI:** 10.6028/jres.108.037

**Published:** 2003-12-01

**Authors:** Rajeev Gupta, Simon G. Kaplan

**Affiliations:** National Institute of Standards and Technology, Gaithersburg, MD 20899-8442

**Keywords:** calcium fluoride, etalon, Fourier transform spectrometer, refractive index, synchrotron, thermal coefficient, ultraviolet

## Abstract

We have constructed a new facility at the National Institute of Standards and Technology (NIST) to measure the index of refraction of transmissive materials in the wavelength range from the visible to the vacuum ultraviolet. An etalon of the material is illuminated with synchrotron radiation, and the interference fringes in the transmittance spectrum are measured using a Fourier transform spectrometer. The refractive index of calcium fluoride, CaF_2_, has been measured from 600 nm to 175 nm and the resulting values agree with a traditional goniometric measurement to within 1 × 10^−5^. The uncertainty in the index values is currently limited by the uncertainty in the thickness measurement of the etalon.

## 1. Introduction

Refractive index is an important physical parameter in the characterization of transmissive materials for optical components for a wide variety of uses, including medical imaging systems, photography, and manufacturing of high quality optical lenses for photo-lithography. Indeed, the required uncertainty of the refractive index for the materials used in a traditional imaging system for photolithography is on the order of 10^−6^ at the wavelengths of interest [1].

Steady growth in the semiconductor industry has been accompanied by the production of integrated circuits (ICs) with ever-shrinking feature sizes. The photolithographic process is one of the key elements in the sequence of steps associated with the manufacture of semiconductor ICs. Photolithography involves exposure of a mask with ultraviolet (UV) light, whereby the pattern on the mask is transferred to a photo-resist. After subsequent processing and etching, the pattern is transferred onto a silicon wafer. The smallest achievable feature size of the patterns is proportional to the wavelength of the light used to expose the mask. Thus, for smaller feature sizes, and hence faster ICs, one would like to use the shortest practical exposure wavelengths. Current manufacturing technology uses excimer lasers operating at 248 nm and at 193 nm for the photolithographic steps. Future efforts to reduce the feature size may involve using F_2_ excimer lasers operating at 157 nm [2].

Most of the designs for photolithographic exposure tools, commonly known as steppers, involve using combinations of high quality lenses and mirrors to transfer the mask pattern onto the photoresist that is spun onto a silicon wafer. To obtain a diffraction-limited pattern one requires high quality optical materials and precision optics. The refractive index, dispersion, and thermo-optic coefficient of the lens materials, commonly fused silica for the 193 nm stepper and calcium fluoride for the 157 nm stepper, have to be accurately determined to allow optical engineers to model the diffraction-limited exposure patterns. A number of measurements of these important quantities have recently been reported [3–6].

## 2. Measurement Technique and Experimental Setup

Numerous approaches have been used to measure the refractive index of transmissive materials with high accuracy. The most common method involves measuring the angle of minimum deviation in a prism constructed of the material whose index is to be determined [7, 8]. This classical method is limited by the accuracy with which the apex angle of the prism and the angle of minimum deviation can be measured. Typically, to obtain an index uncertainty less than 1 × 10^−5^, one requires an angular uncertainty of less than 1″, which poses difficult technical challenges in the construction of a spectro-goniometer.

We have employed an approach that involves the use of a precision optical flat and the observation of interference fringes in the transmission spectra through the sample with the use of a unique high resolution UV Fourier transform spectrometer (UV-FTS). The UV-FTS has the advantage of high throughput, signal-averaging, high resolution, and wavelength accuracy, compared to a traditional grating spectrometer. This method also has the advantage of giving a quasi-continuous spectrum of index and dispersion values, as opposed to values at isolated (and serendipitous) wavelengths from a spectral lamp used with the prism method.

When an etalon sample is illuminated with white light, one observes a set of interference fringes in the spectrum of the transmitted light. Interference maxima will be observed at wavenumbers *ν_m_* given by the formula

$$v_{m}=\frac{m}{2tn\left(v_{m}\right)}$$ (1)

where n (*ν_m_*) is the index of refraction at the fringe maximum, *t* is the physical thickness of the etalon sample, and *m* is the order of the interference fringe. Thus a measurement of the fringe spectrum at high resolution can be combined with a high accuracy measurement of the physical thickness of the sample to yield high accuracy values of the refractive index of the sample over a range of wavelengths.

To measure the refractive index continuously from the visible down to the vacuum ultraviolet, we use the Synchrotron Ultraviolet Radiation Facility III (SURF III) at NIST [9], which provides radiation emitted from electrons confined to move in a circular orbit. Because of the relatively low electron energy and highly uniform magnetic field of the storage ring, SURF III is especially suited for this measurement by providing a uniform, stable beam over the whole spectral range. A new beamline (BL-5) was specifically designed to use the UV-FTS with the synchrotron source.

**Fig. 1:**
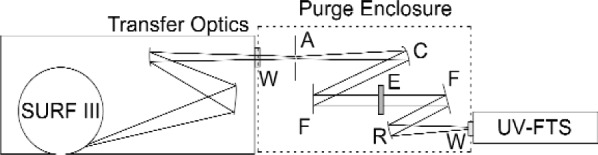
Schematic diagram of the experimental setup used to measure the refractive index, showing the imaging optics used to image the SURF III beam onto an aperture, collimation optics, etalon, re-imaging optics, and the UV-FTS: W, vacuum window; A, aperture; C, collimating mirror; F, fold mirror; E, etalon sample; R, re-focussing mirror. The transfer optics and UV-FTS are housed in vacuum chambers, while the sample and associated optics are placed in a flowing N_2_ purge enclosure.

The beamline consists of imaging optics, which uses two parabolic mirrors (focal length of 1.5 m) to image the 1 mm × 3 mm synchrotron radiation beam at the orbital plane onto an aperture. A schematic diagram of the apparatus is depicted in Fig. 1. A mirror assembly housed in an ultrahigh vacuum chamber focusses the radiation onto the MgF_2_ exit window. An aperture and set of mirrors are used to construct a collimated beam in which the sample etalon is placed. Reasonable collimation (≈f/200) is achieved by retro-reflecting the light back onto the aperture and comparing the size of the beam with the size of the aperture. The etalon is carefully aligned perpendicular to the beam to within ≈1 mrad.

The resultant interference pattern in the transmittance spectrum of the etalon is measured by imaging the collimated beam onto the entrance aperture of the UV-FTS. The beam is made slightly convergent to maximize the coupling of the light into the f/20 acceptance angle of the UV-FTS. Details on the construction and use of the UV-FTS have been reported previously [10]. The spectral measurement of the transmittance is made with two sets of photo-multiplier tube detectors—one for the visible spectral range and a solar blind set optimized for the UV below 250 nm wavelength. The UV-FTS uses a calcium fluoride beamsplitter, which has a short wavelength operating limit of approximately 135 nm. Two retro-mirrors, each consisting of a plane mirror and a parabolic mirror, are used; one retro-mirror is held fixed while the other is scanned to obtain the interferogram (the Fourier transform of the spectrum). The spectrometer has an ultimate resolution of 0.025 cm^−1^, determined by the 0.2 m travel of the moving retro-mirror.

The collimating mirror assembly, sample, refocusing optics, and detectors are housed in sealed enclosures, which are purged with N_2_ gas from liquid N_2_ boil-off at an overpressure of ≈40 kPa. The UV-FTS is operated under vacuum at a pressure of ≈10^−3^ Pa. The ambient temperature is monitored and was in the range of 20 °C ± 1 °C.

## 3. Results

We have measured the refractive index of a 1 mm thick sample of calcium fluoride. The thickness of the etalon was measured by the Precision Engineering Division at NIST using a mechanical contact method, which compares the sample etalon to a gauge block which is measured interferometrically [11]. The thickness is measured at several points on the sample in order to estimate the flatness of the 25 mm diameter etalon. The measurements were made at 20 °C, which corresponds to the temperature at which the measurements of the refractive index were made.

**Fig. 2:**
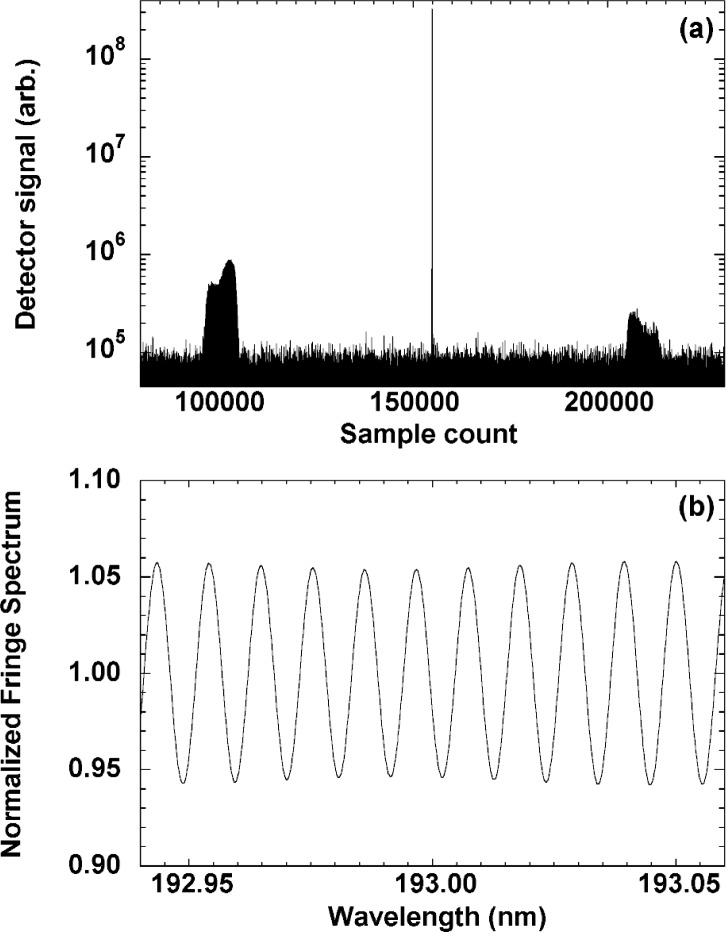
(a) Interferogram acquired with the UV-FTS at a resolution of 0.5 cm^−1^ with a 1 mm thick CaF_2_ etalon sample in the beam, and (b) the ratio of the Fourier transform of the noise-reduced interferogram to the center-burst transform, showing a portion of the normalized fringe spectrum near 193 nm.

Typical flux levels of 10^13^ photons s^−1^ nm^−1^ mA^−1^ were available from the radiation from SURF III and most of the measurements were made with ≈200 mA of electron current in the storage ring. A typical interferogram (40 min collection time) is shown in Fig. 2(a). The center burst corresponds to the zero optical path difference configuration and the side bursts are the result of the optical path difference through the sample etalon. The asymmetry in the side bursts is due to slight misalignment of the interferometer.

The interferogram is transformed to yield a set of fringes as a function of wavenumber as shown in Fig. 2(b). The observed peak-to-peak modulation of 12 % is consistent with a sample thickness variation of ±13 nm over the 20 mm beam diameter. We improve the signal-to-noise ratio of the fringe spectrum by artificially setting the regions between the center burst and the side bursts to zero before transforming the interferogram. Care is taken to set only the values that are below the noise level to zero, so as not to lose any information from the interferogram or add any spurious features to the spectrum. In addition, obvious spikes in the interferogram due to transient electronic noise are removed. Measurements were repeated after realigning the etalon to estimate the sensitivity to small misalignment and beam fluctuations. The relative humidity of the gas surrounding the sample and the temperature of the sample were monitored.

**Fig. 3:**
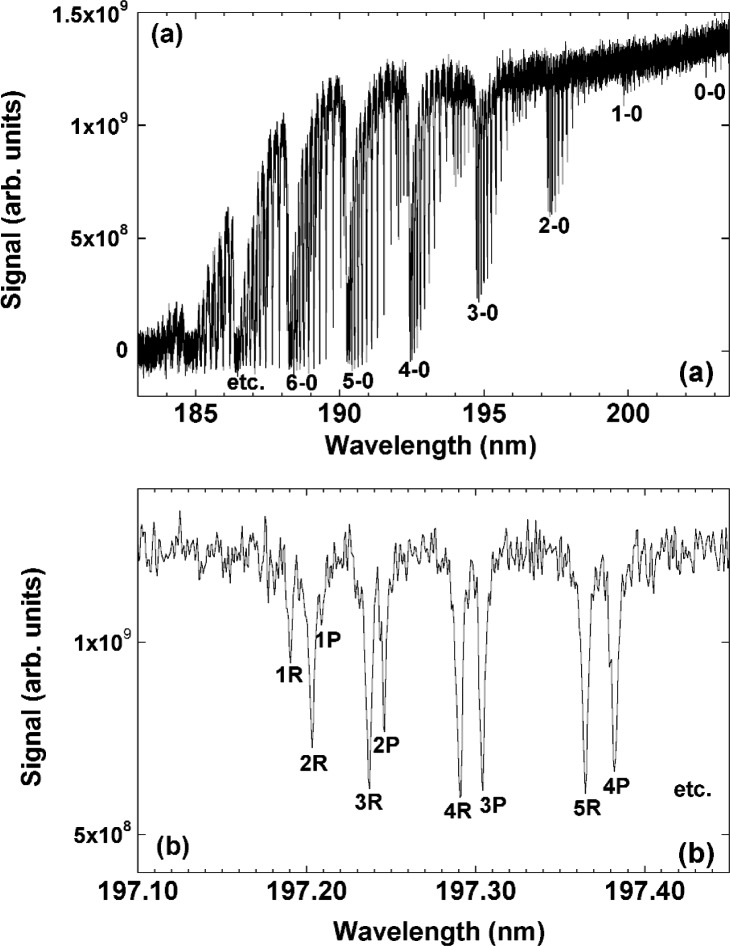
The spectrum of O_2_ absorption lines over the wavelength range from 180 nm to 205 nm, with a resolution of 0.25 cm^−1^ and (b) an expanded view of the 2-0 sub-band around 197.25 nm showing the series of ro-vibrational transitions.

The wavelength scale of the UV-FTS was calibrated using the set of rotational-vibrational sub-bands in the O_2_ electronic spectrum, after introducing air into the purge enclosure around the sample. Because the optical path through the air is essentially identical to that through the N_2_ purge gas in the sample measurement case, this method provides a sufficient calibration without need to configure a standard spectral line source collinear with the synchrotron beam. The wavelength scan of oxygen in air was compared to the tabulated values of the Schumann-Runge band, which has been carefully measured with a high-resolution spectrometer [12]. The oxygen spectrum is shown in Fig. 3(a) over a wavelength range from 182.5 nm to 205 nm, with a resolution of 0.25 cm^−1^. A series of ro-vibrational bands can be seen and one of them (2-0) is shown on an expanded scale in Fig 3(b). The magnified plot shows a set of doublet peaks whose center wavenumbers are plotted against the tabulated values to yield a wavelength correction for the UV-FTS. The residuals from the fits are ≈0.1 cm^−1^ around 50 600 cm^−1^, and the calculated relative wavelength correction is approximately 4 × 10^−6^.

**Fig. 4:**
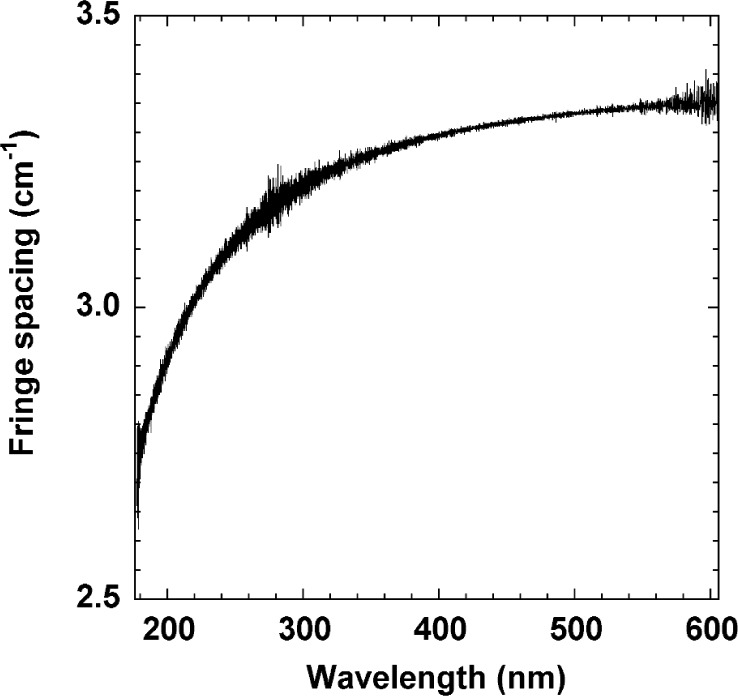
The fringe spacing as a function of wavelength for the CaF_2_ etalon in the wavelength range from 175 nm to 600 nm. Increased dispersion can be seen at wavelengths below 400 nm.

The wavelength corrected sample fringe spectrum is analyzed using a centroid peak-finding algorithm to yield a series of fringe maxima wavenumbers, with an arbitrary initial fringe order *m*. The resulting fringe spacing as a function of wavelength is plotted in Fig. 4. The fringe spacing is fairly constant at wavelengths above 400 nm and decreases rapidly at shorter wavelengths. This decrease corresponds to an increase in the dispersion at shorter wavelengths. The continuous nature of the curve demonstrates the quality of the data, i.e., there are no missing or extra (spurious) peaks, while the fluctuations (≈0.01 cm^−1^ to 0.05 cm^−1^) in the spacing give an indication of the repeatability component of the relative uncertainty in the individual index of refraction values. One could use the data as presented in Fig. 4 to obtain low-accuracy (≈10^−3^ uncertainty) values for the “group” index of refraction by differentiating Eq. (1):

$$\Delta v_{m}≅\frac{1}{2t\left({n\left(v_{m}\right)+v_{m}\frac{dn}{dv}|}_{v=v_{m}}\right)}$$ (2)

where *Δν_m_* = *ν_m_*_+1_−*ν_m_*. However, in order to obtain high-accuracy values for the phase index *n*, one must somehow determine the fringe order *m* in Eq. (1) with 0 uncertainty.

There are several approaches that may be used to obtain *m* and thus the absolute refractive index of the sample [13]. The choice of method depends on the information about the sample available to the user and the goal of the measurement. For most lithographic applications, the materials are fairly common, their index values are well known in the visible, and a high accuracy value in the UV is to be determined. For such applications, one need not make independent determinations of both the thickness of the sample and the fringe order to get a high accuracy value of the index in the UV; accurate knowledge of the index at two sufficiently different wavelengths is enough to determine both *t* and *m* in Eq. (1).

Alternatively, it may not be possible to access high accuracy index values at two known wavelengths, but one value of sufficient accuracy may be available and used to determine the fringe order with zero uncertainty given the sample thickness. The determination of the fringe order at a particular wavelength from the known rough index value along with the sample thickness can be used to get a high accuracy value of the index at any other wavelength. The limiting factor in the index determination with this approach is usually the accuracy of the (mechanical) thickness measurement.

Yet another approach may be used in the instance where there is no prior information about the refractive index. In this case, one may get an initial determination of the refractive index from an accurate (≈10^−3^ uncertainty) low-resolution measurement of the sample reflectance and/or transmittance [13]. For thin samples, this measurement can yield a value for the fringe order and a refined value of the index can be calculated from the fringe spectrum. This higher accuracy refractive index value may be used to determine *m* for a thicker sample, and the process can be repeated. Alternatively, a comparison of samples of different thicknesses can be used as a “vernier scale” to fix the fringe order for both samples. Since both the fringe orders must be integers, by examining two fringes in each sample that are close enough together that the index must be negligibly different for the two positions, one can deduce the fringe orders for both samples.

Finally, in some cases there may be enough information about the functional form of the refractive index that Eq. (1) or (2) can be used to fit the data and extrapolate to a wavenumber where *m* is known (0 cm^−1^, for instance). This method has been applied to Si in the infrared transparency region [14].

We measured the refractive index of UV grade calcium fluoride (acquired from Crystal Saint-Gobain^1^) by the traditional prism method at two wavelengths (508 nm and 581 nm) in the visible. These index values were used to determine the thickness and fringe order in the visible and thus high accuracy index values at all other wavelengths. A value of 1.501 917 was determined at the excimer wavelength of 193.39 nm, which was found to be in excellent agreement with the published value for calcium fluoride (20 °C) of 1.501 930 [3]. The agreement is within the uncertainty of the tabulated values in the visible and in the UV. The optically determined thickness value of the etalon sample of 1.030436 mm ± 0.000006 mm agrees with the mechanical measurement result of 1.030460 mm ± 0.000028 mm (*k* = 1).

**Fig. 5:**
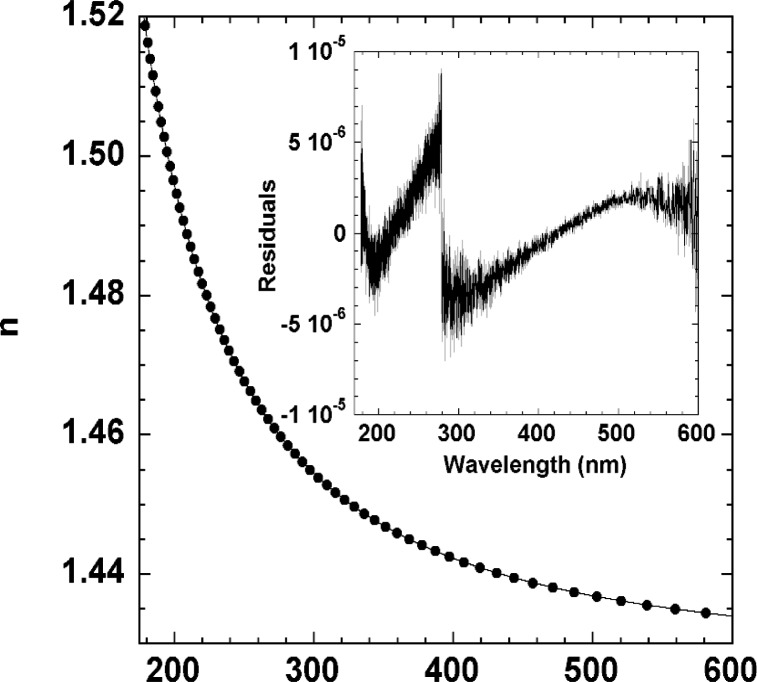
Refractive index values of CaF_2_ plotted as a function of wavelength in the range from 175 nm to 600 nm (symbols), along with the fit to Eq. (1) as described in the text (solid line). The differences between the measured values and the Sellmeier fit are shown in the inset.

In order to further reduce the noise and produce a compact representation of the refractive index results, we fit the index spectrum to a three-term Sellmeier formula

$$n\left(\lambda \right)=\sqrt{1+\frac{S_{1}\lambda^{2}}{\lambda^{2}-{\lambda_{1}}^{2}}+\frac{S_{2}\lambda^{2}}{\lambda^{2}-{\lambda_{2}}^{2}}+\frac{S_{3}\lambda^{2}}{\lambda^{2}-{\lambda_{3}}^{2}}}$$ (3)

where we fix *S*_3_ = 3.85 × 10^−6^ and *λ*_3_ = 34 600 nm (taken from handbook values for CaF_2_ [15]). The remaining four parameters are determined from a nonlinear least-squares fit to the ≈13000 data points. The resulting values are *S*_1_ = 6.618 941 × 10^−7^, *λ*_1_ = 55.71663 nm, *S*_2_ = 3.778733 × 10^−7^, and *λ*_2_ = 104.5846 nm. The solid line in Fig. 5(a) shows the fit to Eq. (3), while the inset shows the residuals, which are of the order ±5 × 10^−6^. The discontinuity at 280 nm is likely due to a temperature drift between the visible and UV measurements. Overall, the difference between the fit values and the experimental results are smaller than the estimated experimental uncertainty discussed below, and the fit should provide somewhat better values near the ends of the spectral range, where the noise in the fringe positions becomes significant.

## 4. Uncertainty Analysis

The sources of uncertainty in the quantities in Eq. (1) which are used to determine the index of refraction can be analyzed and used to produce an estimate of the uncertainty in *n*. Table 1 lists the major sources of uncertainty, along with their estimated values for a temperature of 20 °C and a wavelength of 193 nm. Each uncertainty source is identified by its type (A for statistically evaluated, B for otherwise). Since the index of refraction is calculated as a linear ratio or product of these measured quantities, the relative standard uncertainty in *n* due to each uncertainty source is simply the relative standard uncertainty in each measured quantity (shown in the rightmost column.) The combined relative uncertainty and resulting standard uncertainty (coverage factor *k* = 1) are then calculated. The value of 1.3 × 10^−5^ is fairly representative of the uncertainty in n over the full spectral range of the reported measurements.

## 5. Conclusions

We have derived high accuracy refractive index values for calcium fluoride over the wavelength range from 600 nm to 175 nm from a measurement of the interference fringes in the transmittance spectrum of a high quality etalon sample. The continuum source radiation from SURF III is analyzed using a custom-built UV-FTS. Excellent agreement was obtained between the index values determined from this technique and the traditional prism-goniometer technique at the excimer wavelength of 193.39 nm.

In the future we would like to extend our measurements of the refractive index to shorter wavelengths and also develop a technique for measurement of the thermal coefficient of the refractive index. Work is currently underway to extend this interferometric technique for the measurement of refractive indices of liquids. This technique is well suited for liquids that are not highly transmissive and are to be used in thin layers for applications such as immersion lithography.

## Figures and Tables

**Table 1:** Uncertainty budget for index of refraction measurement of 1 mm thick CaF_2_ etalon sample at 193 nm

Uncertainty Source	Type	Standard uncertainty	Rel. std uncertainty in *n*
Thickness measurement (nm)	B	6	5.83E−06
Angle of incidence (°)	B	0.12	2.19E−06
Beam divergence (°)	B	0.15	4.00E−07
Temperature (K)	B	0.5	5.75E−06
Wavenumber error in UV-FTS (cm^−1^)	B	0.1	1.93E−06
Peak center determination (cm^−1^)	A	0.02	3.86E−07
Fringe order	B	0	0.00E+00
Intrinsic birefringence (10^−6^)	B	0.34	3.40E−07
Quadrature Sum			8.72E−06
Uncertainty in *n* (*k* = 1)		1.31E-05	

## Footnotes

1 Certain commercial equipment, instruments, or materials are identified in this paper to foster understanding. Such identification does not imply recommendation or endorsement by the National Institute of Standards and Technology, nor does it imply that the materials or equipment identified are necessarily the best available for the purpose.
